# Supplementary material for: Are carbon emissions trading and green financial instruments synergistic? -Comprehensive quantitative research based on content analysis
Source: PLoS One. 2024 Mar 7;19(3):e0298601. doi: 10.1371/journal.pone.0298601 (PMC10919601; doi:10.1371/journal.pone.0298601)
Supplement: S1 Data — (ZIP) [file pone.0298601.s001.zip › Data/地级市Z_F工作报告.zip!/╣π╢1⁄2/╔╟╬▓╩╨/└┤╫╘╣1⁄2╓┌║┼í╛┬φ┐╦╩2╛▌═°í┐_2019.docx]

各位代表：

我代表市人民政府向大会作政府工作报告，请予审议，并请政协各位委员和其他列席人员提出意见。

2018年工作回顾

刚刚过去的2018年，是全面贯彻党的十九大精神开局之年，是改革开放40周年、汕尾建市30周年。我们坚持以习近平新时代中国特色社会主义思想为指导，按照党的十九大作出的战略部署，高举新时代改革开放旗帜，深入学习贯彻习近平总书记对广东重要讲话精神，落实省委李希书记在汕尾调研时讲话精神，按照省委、省政府和市委的工作部署，坚持稳中求进工作总基调，贯彻新发展理念，落实高质量发展要求，统筹推进稳增长、促改革、调结构、惠民生、防风险各项工作，推动经济社会持续平稳健康发展，顺利完成市七届人大三次会议确定的目标任务。

综合实力稳步提升。预计实现地区生产总值（含深汕特别合作区）951亿元，增长8%；三次产业结构调整为14.5:42.8:42.7；规模以上工业增加值223.9亿元，增长11%，先进制造业、高技术制造业增加值占规上工业增加值比重分别提高到32%、25.9%；固定资产投资641.1亿元，增长18%；社会消费品零售总额611亿元，增长9.8%。一般公共预算收入42.3亿元，增长15%，增速居全省第1位。

发展后劲不断增强。深汕特别合作区体制机制调整后正式挂牌，交出探索区域协调发展的最新答卷，深汕一体化全面提速。省出台《海陆丰革命老区振兴发展规划》，59项扶持政策含金量十足。广汕高铁建设顺利推进，汕汕高铁、深汕西高速改扩建工程动工建设。汕尾高新区扩区获省批准。陆河比亚迪年产值超百亿，信利TFT5代线正式投产，宝丽华甲湖湾电厂并网发电，汕尾海上风电项目正式动工，海工装备基地落户陆丰，一批层次高、支撑力强的骨干企业发挥效益，电子信息、新能源汽车、电力能源等主导产业不断壮大。第四次全国经济普查清查有效单位2.2万家，比上次普查增加1.4万家、增长175%；个体经营户10.8万家，增加2.8万家、增长35%。

城乡环境更加宜居。城市品质不断提升，中心市区建成面积33平方公里，较三年前拓展近一倍。香江大道（汕马路）、海滨大道中、红海湾大道、汕尾大道中（西闸至埔边）等4条城市主干道，红海东路等7条市政道路，金鹏路西等10条断头路综合改造加快推进。美丽宜居乡村建设全域推进，城区晨洲、海丰富足园、陆河螺洞、陆丰吉水等一批样板村各具特色、各美其美，海丰新山、坡平、陆丰下埔、陆河湖坑、红海湾东尾等8个省定“红色村”旧貌换新颜。

民生福祉持续增进。财政用于民生的支出195.8亿元，占一般公共预算支出比重达77.7%。省、市民生实事37项工作任务进展顺利。城镇登记失业率2.35%，城镇新增就业5万人，转移农村劳动力2.98万人，城镇居民、农村居民人均可支配收入分别增长7.2%、9%。中山大学深汕中心医院桩基工程基本完成，医师规培、学科建设等前期工作逐项推进，市第三人民医院、9家县公立医院、6家民营医院加快建设，36家乡镇卫生院、175家村卫生站标准化规范化建设基本完成，新增病床1395张。新改建幼儿园94所、中小学校10所、职业学校3所，新增学前、义务教育学位2.6万个。

发展环境显著改善。全市上下众志成城、雷霆扫毒，陆丰毒品问题得到根本解决，成功摘除“毒帽”。碣石洋垃圾问题得到彻底根治，经验做法得到全国打私办肯定。乌坎土地问题全面解决。全市实现“四个不发生”，刑事立案数全省最低，万人犯罪率不到全省平均水平一半。提前接受创建省文明城市测评。营商环境不断优化，审批效率明显提升，新开办企业仅需4天。环境空气综合质量指数连续4年稳居全省第1位。

多项工作提档进位。在前几年扎实工作、取得成效的基础上，我们再接再厉，推动多项工作再上新台阶。各项主要经济指标增幅均名列全省前茅，政府公共服务总体满意度跃升至全省第10名、粤东西北第2名。打假考核跃升至全省第12名。保障农民工工资支付考核全省第1名。食用农产品快检考核跃升至全省第3名，食品安全考核跃升至全省第13名。粮食安全考核跃升至全省第12名。污水处理设施考核跃升至全省第12名。成功创建国家社会保险标准化先行城市，智慧人社建设走在全省前列。

一年来，我们主要做了以下工作：

（一）及时出台有效政策措施，全力促进经济加快发展。认真贯彻中央、省宏观调控政策，坚持超前研判、精准施策、要素保障、链式推进，深化供给侧结构性改革，实现经济快速增长。大力降低实体经济成本，出台市“实体经济新十条”，推出58项政策措施，预计全年为企业新减负约15亿元。出台支持民营经济发展举措，预计民营经济增加值增长8%。制订出台市“外资新十条”，进一步强化投资贸易便利化措施，整体通关时间压缩1/3，实际利用外资6.9亿元、增长3%，总量位居粤东西北前列。出台“促进就业九条”，全力保障企业用工，帮助企业解决用工11692人。推进产业园区提质增效，投入园区建设资金10.2亿元，加快征地及路水电气讯等配套设施建设。开展新一轮大招商行动，推动产业项目落地建设，红草比亚迪新能源汽车零部件和消费类电子、国润纺织等24个产业项目正式投产，威翔航空、路华电子等34个项目动工建设，深投控、秋叶原等43个项目落户园区。推进工业转型升级，完成工业投资252.3亿元，其中技改投资94.3亿元。加快农业发展，实现农业总产值223.4亿元，增长5.5%。金融机构各项存款余额、贷款余额分别增长12.4%、4.3%。接待过夜游客、旅游总收入分别增长9%、22%，新增2家国家4A级景区。

（二）持续深化改革创新，发展活力不断释放。坚持以改革为动力、以创新为引领，不断增强经济高质量发展的战略支撑力。扎实推进市县机构改革，市级机构改革进展顺利。突出重点领域改革和基层改革探索创新，扎实推进“放管服”改革十项重点任务，清理规范中介服务事项19项，取消各类证明240项。推进“数字政府”建设，实现政务服务“一网通办”。全面实施“多证合一”，新登记各类企业增长61%，各类市场主体达到16.9万户，新增“四上”企业97家，完成“个转企”1514户。搭建国有住房租赁平台，筹建市属国有资本运营公司。深入实施创新驱动战略，预计R&D经费占GDP比重0.94%。新增国家高新技术企业14家，培育入库科技型中小企业36家。省级重点实验室、省级博士后创新实践基地均实现零的突破，新建省级工程技术研究中心6家。获省科技进步奖1项，PCT专利申请11件。专利申请量、专利授权量分别增长88%、200%，增幅居全省第3位、第1位。深入实施红海扬帆人才计划，引进45名高层次人才。

（三）加快补齐基础设施短板，城市建设日新月异。发挥有效投资关键作用，完成24个省重点项目投资79.3亿元、186个市重点项目投资323.8亿元。投入资金24.2亿元，推动铁公机港等交通基础设施建设，深汕高铁、龙汕铁路前期工作进展顺利，兴汕高速主体线形基本成型，广东滨海旅游公路汕尾段、珠东快速前期工作有序推进，新改建普通公路135.4公里，启动汕尾至香港海上客运航线复航规划研究，陆丰甲湖湾电厂10万吨级配套码头建成使用，小漠港2个10万吨、1个5万吨码头泊位启动沉箱作业。赤沙水厂、公平灌区节水改造工程加快建设，29宗、343公里中小河流治理和一批大中型水闸除险加固工程稳步推进。完成电力、通讯基础设施投资18亿元，甲湖湾电厂送电线路工程全线贯通，489个村完成电网改造，新建4G基站588座，新增光纤用户7.9万户，光纤入户率达89.5%。高标准启动“多规合一”城市空间规划及县域新一轮城市总体规划修编。畅通城市交通微循环，拓宽优化香洲路、红海中路、文明路等一批市政道路，滨海大道西及沿线景观规划设计完成国际招标。保利大都汇、万升广场、农商行大厦、碧桂园、恒大、星河湾、雅居乐等一批城市地标性建筑加快建设。品清湖蓝色海湾整治扎实推进。以决战决胜姿态冲刺“创文”“创国卫”，推进市县镇村四级文明联创，持续开展“十大整治、十大提升”行动，环境卫生、交通秩序、农贸市场、户外广告得到整治，治理违建65万平方米，其中拆除42.5万平方米，市区非法运营三轮车整治成果得到巩固，新增公交线路7条，公交准点率提高至95%。中心县城扩容提质亮点纷呈，海丰海丽大道一期、海紫路完成升级改造，建成2.7公里红色文化街；陆河大道、陆河人民北路延伸段相继建成通车，21条市政道路和螺河两岸完成升级改造；陆丰国道324线穿城段、东河两岸完成升级改造，青少年宫等市民活动场所加快建设。

（四）扎实推进三大攻坚战，三年行动开局良好。有效防范化解金融风险，依法严厉打击三家农信社逃废债行为，立案133宗，拘留或逮捕涉嫌违法犯罪人员88名，追回不良贷款36.3亿元，海丰、陆丰、陆河农商行筹建申请获批。政府综合负债率仅为17%，债务余额全省最少。坚持“真扶贫、扶真贫、真脱贫”，推进资产收益扶贫、产业就业扶贫、政策兜底扶贫，实施产业扶贫项目1.9万个，实现2.1万贫困人口脱贫，基本完成贫困户危房改造。深圳市累计投入新一轮帮扶资金39.2亿元，帮扶到村项目1958个、到户项目9.4万个，成功打造陆河华侨城螺溪谷、陆丰下埔万科红村碧海等一批精品项目。坚决打好污染防治攻坚战，全力以赴抓好中央环保督察“回头看”、省环保督察问题整改，在反馈意见中我市获得好评。率先在全省完成地级饮用水水源地环境问题“清零”，大湖鸟类自然保护区内14家高位养殖场全部拆除，完成陆丰、海丰生活垃圾简易焚烧问题整治，基本完成奎山湖、奎山河黑臭水体整治。集中式饮用水源地、中型以上湖泊水库、近岸海域水质达标率100%。全面落实河长制、湖长制，清理河道1394公里。陆丰东南生活垃圾焚烧发电厂建成运营，市生活垃圾无害化处理中心二期主体完成建设，启动市医疗废物集中处置中心建设，与华润电厂合作推进污泥处置，碣石、螺溪、梅陇等7个中心镇污水处理厂通水运行，新建污水管网162公里，城镇生活垃圾无害化处理率、生活污水处理率达92.7%、88.5%。推进新一轮绿化大行动，创新推行“山长制”，森林火灾数和过火面积分别下降79%、74%，连续三年取得清明森林防火攻坚战全面胜利。万众一心成功抗击陆河“8·31”特大水灾和台风“山竹”。

（五）坚持农业农村优先发展，乡村振兴战略扎实推进。高质量推进全域美丽乡村建设，142个省定贫困村创建新农村示范村工作成效明显，完成省级补助资金支出13.13亿元，支出率居全省前列。首批286个非贫困村完成村庄规划编制、基本完成人居环境整治。完成生活垃圾、生活污水、水体污染整治6234宗，绿化美化59.2万平方米，完成农村道路硬化1269公里，改造农村危房851户，改建农村无害化户厕354座。大力发展现代农业，创建海丰蔬菜、陆丰萝卜2个省级农业产业园，新增省重点农业龙头企业4家、省级农业标准化示范区6个、省级名牌农产品2个、农民专业合作组织1648家，复耕撂荒土地7.67万亩。与省农科院合作共建汕尾分院，海丰、陆河被评为省电子商务进农村综合示范县。省委、省政府在我市召开全省抓党建促乡村振兴暨红色村党建示范工程现场会，海丰新山村“两个示范”建设成效喜人，国庆节以来接待游客超20万人。农村土地确权登记颁证工作基本完成，集体产权制度改革试点工作稳妥推进。

（六）持续改善民生事业，群众获得感显著增强。市本级财政下拨10.2亿元支持基层建设，其中城乡养老等配套补助6.6亿元，美丽乡村建设补助1.43亿元，禁毒经费4000万元，乡镇综合治理经费5700万元，村创文经费4400万元，村活动场所建设经费5600万元。争取国家、省各项转移支付资金155亿元，获批政府债券53亿元。《海陆丰革命老区振兴发展规划》政策红利初显，仅省“三个全额负担”和县级财力补助减轻负担10.72亿元，其中减免广汕高铁资本金7.54亿元、义务教育公用经费1.1亿元、基层医疗卫生建设资金1.28亿元，增加县级财力补助8000万。五大险种累计参保人次达549.5万人。深圳帮扶的城区田家炳中学、盐町头小学、华侨中学等3所学校和陆河东坑等6家乡镇卫生院投入使用，市级教师技能实训中心、市健康医疗数据中心动工建设。城区、陆河、红海湾、华侨成功创建省推进教育现代化先进县（区）。市高级技工学校加快建设。取缔无证幼儿园245所，撤并“麻雀学校”84所。新增名校长、名教师、名班主任工作室25个，实现全市57个镇中心学校少年宫全覆盖。组建城区医疗集团、市人民医院医疗集团等医联体。加强食品药品市场监管。完成3间福利院、34间敬老院升级改造。举办广东（汕尾）红色文化旅游主题系列活动，汕尾渔歌、西秦戏晋京展演，白字戏《公烛》获省群众戏剧曲艺花会金奖。举办市第一届老年人体育健身大会。

（七）加强和创新社会治理，社会大局保持和谐稳定。深入推进平安汕尾建设，社会安全稳定形势持续向好。扫黑除恶工作成效位居全省前列，侦破涉黑涉恶案件517宗，打掉涉黑涉恶团伙114个，抓获1339人，刑拘1249人，得到中央扫黑除恶督导组充分肯定。开展“飓风2018”专项行动，推进社会治安防控体系“铸盾”行动，全市260天“零双抢”。连续17年未发生重特大生产安全事故。开展信访积案集中攻坚，办结省领导包案2宗、市县领导包案48宗，办结率分别达到100%、77.4%。社会矛盾纠纷调解成功率99.9%，进京上访人次下降40%。完成10个村（社区）创新城乡社区治理专项改革先行点建设。推动志愿服务融入基层社会治理，新增注册志愿者4.7万人、志愿服务团体799个。高质量完成国防动员演练，扎实做好优抚安置和双拥工作。

此外，民族、宗教、对台、侨务、人防、气象、慈善、档案、方志等工作取得新进步。

过去一年，我们扎实推进政府自身建设，深入开展“大学习、深调研、真落实”活动。认真执行市人大及其常委会决议决定，定期向市人大常委会报告工作、向市政协通报情况，自觉接受监督，办理人大代表建议39件、政协提案138件，办复率100%。《汕尾市革命老区红色资源保护条例》获准施行。推进法治政府建设，落实行政机关负责人出庭应诉制度，改变了“告官不见官”的局面，法治汕尾迈出一大步。全面推动政务公开，开展宪法宣教活动。落实党风廉政建设主体责任，坚决全面彻底肃清李嘉、万庆良恶劣影响，不折不扣抓好中央巡视整改任务落实，扎实推进廉政风险防控。严格落实中央八项规定及实施细则精神，加大审计监督力度，严控“三公”经费，政府作风进一步转变。

各位代表，过去一年的成绩来之不易，这是以习近平新时代中国特色社会主义思想为指导的结果，是省委、省政府和市委坚强领导的结果，是全市人民凝心聚力、团结奋斗的结果，是深圳等兄弟市和海内外各界人士大力支持的结果。在此，我代表汕尾市人民政府，向全市人民、驻汕部队官兵、各民主党派、人民团体、社会各界人士，向长期关心支持汕尾改革发展的港澳同胞、台湾同胞、海外侨胞，表示崇高的敬意和诚挚的感谢！

我们也清醒认识到，汕尾发展中还存在不少困难和挑战。一是高质量发展任务艰巨。经济发展总量、均量、质量有待提升，规上工业企业数全省最少，规上工业增加值占全省比重不足百分之一，产业结构总体处于中低端，创新引领能力不强，传统产业转型升级有待加快。实体经济发展面临不少困难，支持实体经济发展的政策措施、营商环境需进一步加强和改善。二是城乡发展仍不平衡。城市化水平相对偏低，中心城区首位度、人口吸引力不足，道路交通、地下管网等基础设施欠账多，停车难问题突出，中心镇缺乏系统规划，农村空心化、耕地撂荒等现象较为突出，城乡融合发展仍需继续发力。三是基本公共服务短板突出。学前教育、义务教育优质学位短缺，基础教育质量亟待提高，职业教育发展滞后。医疗卫生资源总量少、水平低，基层医疗服务能力不强。尚未建成市级社会福利院、文化馆和博物馆，基层文化设施达标率较低。保障房供需矛盾突出。四是社会治理压力较大。影响社会稳定的风险隐患仍然不少，社会矛盾纠纷依然易发、多发，社会治安综合治理手段落后。食品药品安全、安全生产还存在薄弱环节。五是政府职能转变还不到位。部分政策特别是激励政策落实还不到位，政府办事效率有待提高、流程有待优化。政府工作人员的法治思维、专业素养、开放意识需要进一步提升。对于这些问题，我们要采取有力措施加以解决。

2019年工作安排

2019年是新中国成立70周年，是全面建成小康社会收官之战的关键一年，粤港澳大湾区建设加快实施，省委赋予汕尾建设成为沿海经济带靓丽明珠的新定位，汕尾迎来了前所未有的重大历史机遇。尽管经济下行压力增大，但是我们有最近几年投资、产业、项目的良好储备基础，只要保持战略定力、主动作为、驾驭时势，就一定能化危为机、转危为安、乘势而上，奋力走出一条新时代革命老区高质量发展的新路子。

今年政府工作的总体要求是：高举习近平新时代中国特色社会主义思想伟大旗帜，全面贯彻党的十九大和十九届二中、三中全会以及中央经济工作会议精神，全面贯彻落实习近平总书记对广东重要讲话和对广东工作一系列重要指示精神，牢固树立“四个意识”，坚定“四个自信”，坚决做到“两个维护”，按照“四个走在全国前列”、当好“两个重要窗口”要求，统筹推进“五位一体”总体布局，协调推进“四个全面”战略布局，认真落实省委“1+1+9”工作部署、省委十二届六次全会和市委七届八次全会决策部署，坚持稳中求进工作总基调，坚持新发展理念，坚持推动高质量发展，坚持深化改革开放，统筹做好稳增长、促改革、调结构、惠民生、防风险、保稳定各项工作，进一步做好“六个稳”，提振市场信心，打好三大攻坚战，着力提高发展平衡性和协调性，深度融入粤港澳大湾区，打造沿海经济带靓丽明珠，为全面建成小康社会收官打下坚实基础，以优异成绩庆祝中华人民共和国成立70周年。

今年经济社会发展主要预期目标是：地区生产总值增长7%；规模以上工业增加值增长8%；固定资产投资增长15%以上；社会消费品零售总额增长9%；进出口总额增长3%；一般公共预算收入增长7%；城乡居民收入增长7.8%；居民消费价格涨幅控制在3%以内；城镇登记失业率控制在3%以内；节能减排约束性指标完成省下达的年度计划。

为了实现上述目标，重点做好以下十方面工作：

一、全面融入粤港澳大湾区建设，打造沿海经济带靓丽明珠

建设粤港澳大湾区是习近平总书记亲自谋划、亲自部署、亲自推动的国家战略。省委把建设大湾区摆在统揽全局、提纲挈领的核心地位，提出举全省之力全域参与大湾区建设。汕尾是两个紧邻粤港澳大湾区的沿海城市之一，加上深汕特别合作区因素的强大推力，我们有条件在融入粤港澳大湾区建设上走在前列，争当融入粤港澳大湾区先行市。

省委高瞻远瞩提出构建“一核一带一区”区域发展新格局，要求各地充分发挥比较优势，由原来单一发展模式的“跟随者”转变为各自功能的“引领者”，强调把贯通东西两翼与珠三角、覆盖14个市的沿海经济带打造成为全省发展主战场。在这一新格局中，汕尾被省委赋予重任、寄予厚望，我们要抓住机遇，更好更快发展，为全省多作贡献，打造沿海经济带靓丽明珠。

深汕特别合作区体制机制调整后重新挂牌，由深圳全面主导建设，相当于把特区搬到汕尾家门口。我们要主动对标对接深圳，学习特区精神和深圳的理念、效率，接受深圳和合作区的辐射带动，把汕尾发展与深圳、合作区发展定位紧密结合，与深圳东进战略和产业协同共建的需要紧密结合，对汕尾工作进行再定位、再谋划，精准对接、同频共振、全面融入。

争当融入粤港澳大湾区先行市、打造沿海经济带靓丽明珠是汕尾发展的战略选择和根本出路，我们要系统谋划，举全市之力推动落实。

——争当深度融入大湾区现代产业体系先行市，成为大湾区制造业生产基地和产业转移主承接区。对标大湾区构建以加快发展先进制造业、加快传统产业转型升级、培育壮大战略性新兴产业、建设国际金融枢纽、构建现代服务业体系为重点的现代产业体系，按照我市现有产业基础，择取电子信息、新能源汽车、电力能源及装备制造、生物医药、智能制造、海洋经济、新材料等七大产业作为主攻方向，谋划布局23条重点产业链，通过龙头企业引进、产业运营商引进、专业研发平台引进、中介服务机构引进等方式，主动对接大湾区优势企业，深化“总部在大湾区、生产基地在汕尾”“前店后厂”等产业协同共建模式，接受以深圳为主的大湾区城市产业外溢辐射带动，加快构建高质量现代产业体系。

——争当深度融入大湾区国际科技创新中心先行市，成为大湾区协同创新延伸区和创新成果转化承载地。加快推动创建国家级、省级高新区，打造区域创新重要节点。积极融入大湾区创新体系，主动与广州、深圳等城市开展协同创新，尝试“基础研究+研发设计在大湾区、试验+生产在汕尾”合作模式，加强技术、资本、人才等创新要素对接，推动大湾区科研成果在汕尾转化。积极参与大湾区核心技术攻关，争取共用共享大湾区重点实验室等科研资源，力争在新一代信息技术、高端装备制造、绿色低碳、生物医药、新材料、海洋经济、现代种业和精准农业等重大领域形成研发技术集群。积极引进港澳高校资源，探索与港澳建立创新创业基地。

——争当深度融入大湾区现代化基础设施体系先行市，成为大湾区东进门户和互联互通的枢纽节点。抓住深圳投入千亿元以上建设高铁、高速公路、城际线等重大交通基础设施，与深汕特别合作区形成“半小时、一小时”通勤的重大机遇，全面对接大湾区基础设施，加快构建4条铁路、9条高速、2条城际线、2条水上客运、3个港口、1个机场的“陆、海、空”立体交通格局，形成与大湾区“半小时、一小时、两小时”交通圈。

——争当深度融入大湾区优质生活圈先行市，成为沿海高品质生活示范区和大湾区“后花园”。充分发挥空气、水等生态环境优势和滨海城市的特质，完善旅游基础设施与配套服务，引进高端休闲旅游综合体项目，构建休闲度假、养生保健、邮轮游艇等多元旅游产品体系，携手大湾区城市共同打造“一程多站”旅游精品路线，把汕尾打造成大湾区的“后花园”。发挥农业资源丰富的优势，大力发展生态农业、有机农业、都市农业等现代农业，打造大湾区“菜篮子”“米袋子”和“果盘子”。积极引进粤港澳优质教育、医疗卫生资源，实施更优人才引进政策，强化人文交流，完善社保制度衔接，深化社会治理合作，构建与大湾区对接靠拢的高品质公共服务体系。

——争当深度融入大湾区世界级城市群建设先行市，成为深莞惠+汕尾河源“3+2”经济圈副中心城市和大湾区世界级城市群的重要组成部分。汕尾毗邻大湾区，是深莞惠+汕河“3+2”经济圈副中心城市，我们要提升城市功能，主动承接大湾区城市功能疏解、产业转移和人口转移，推动汕尾与大湾区城市群有机融合，推动形成产业联动、交通互通、政策衔接、公共服务匹配、发展环境优化等五个一体化，使汕尾成为大湾区世界级城市群的重要组成部分。

——争当深度融入大湾区全面开放新格局的先行市，成为大湾区全面开放的有机组成部分和营商环境高地。对标最高最好最优营商环境标准，深化“放管服”改革，加快“数字政府”建设，进一步降低制度性交易成本，推动市场准入政策、市场执法标准、市场法治环境、产业扶持政策和产业服务平台实现基本统一，促进人力、物流、资金流和信息流无障碍流动，营造富有弹性的制度环境和营商环境，实现与大湾区机制“软联通”。对标大湾区出台更加优惠的便利措施，推进投资贸易自由化便利化，推动人员车辆货物往来便利化，营造更具竞争力、更加开放透明的投资环境。对外商投资企业同等适用产业支持创新支持等配套政策，争取在引进外资方面取得突破。积极参与“一带一路”建设，深化与沿线国家合作交流。复制推广广东自贸试验区制度创新经验，探索推进开放型经济新体制建设，打造高水平对外开放门户。

二、大力发展实体经济，推动产业高质量发展

——推动制造业集聚发展。高起点谋划发展海岸经济，沿海岸线自东向西从陆丰湖东至马宫、鲘门串珠成链布局现代产业。打造两千亿级投资的电力能源及装备产业，加快陆丰核电6台机组建设，推进500万千瓦海上风电建设，加快海工装备基地建设，形成集技术研发、设备制造、建设安装、运行维护为一体的全产业链。推动信利等龙头企业引进上下游配套企业，完善“材料－面板－模组－整机”纵向产业链。支持比亚迪汽车整车、汽车零配件、电池、新材料、消费类电子等项目增资扩产，促进上下游企业配套集聚，打造新能源产业基地。推动海王、香雪、江涛等医药企业投产，提高生物医药产业化水平。加快建设梅陇首饰、可塘彩宝、公平服装、甲子五金等特色产业集聚地。实施质量强市战略，鼓励更多企业争创名牌和参与制订修订行业标准。

——促进现代服务业壮大发展。重点发展商贸物流、现代金融、电子商务等生产性服务业，加快发展旅游、康养等生活性服务业，带动现代服务业发展提速、比重提高、质量提升。依托电子信息、新能源汽车等主导产业，建设一批生产性服务业公共服务平台，推动生产性服务业与制造业融合发展。促进消费供给升级，推进城市综合体、农贸批发市场和乡镇商贸中心建设，持续挖掘汽车、家电等传统消费潜力，发展文化创意、体育健身等服务消费。推动电子商务进农村，扩大农村消费市场。整合“红、蓝、绿、古、特”旅游资源，开展旅游康养大招商，引进几个大的旅游康养开发项目。做好全域旅游规划，打造一批全域旅游示范区、示范点。大力发展红色旅游、乡村旅游、滨海旅游，推进红海湾滨海旅游度假区、红宫红场、莲花山、玄武山等景区景点优化提升。加快现代旅游标准化建设，推进精品景区建设，全面提升景区建设和管理水平，推动金町湾旅游度假区创建国家4A级景区。做好羊蹄岭、三溪等古驿道保护性开发利用。

——加快现代农业优化发展。推进海丰蔬菜、陆丰萝卜产业园建设，新增省级现代农业产业园3个以上。加快培育一批新型农业经营主体，新增省、市级农业龙头企业8家以上。加大农业品牌建设政策扶持，支持皇斋虎噉金针菜等一批农业品牌做大做强，培育莲花山茶、陆河青梅、华侨红杨桃等一批省级名特优新农业品牌。大力培育发展农产品精深加工、冷链物流、休闲农业、农村电商等新业态，延长产业链、提升价值链。发展粮食产业，确保粮食安全。发挥省农科院汕尾分院平台作用，加强农业科研攻关、技术推广、成果转化和人才培养，增强农业科技支撑力。发展壮大海洋渔业，建设汕尾（马宫）渔港经济区，规划建设市级海产品交易中心和陆丰碣石智慧渔业科创园，加快汕尾渔港、遮浪渔港、甲子渔港、碣石渔港建设。发展远洋捕捞。推动传统养殖转型升级，扶持壮大国泰、五丰、银鹏等龙头企业，深化水产品加工流通。

——激发各类市场主体活力。落实省、市“实体经济新十条”，推进更大力度的减税降费。推动民营经济健康发展，落实省“民营经济十条”，在准入、融资、招工、用地等方面给予民营企业更大支持。发挥粤科金融作用，为园区企业提供全方位的金融服务。落实促进创业各项补助政策，全面优化补贴申领流程。加大“四上”企业培育力度，推动“个转企”，扎实做好第四次全国经济普查工作。倡导崇尚技能、精益求精的“工匠精神”，培育一批技能高超、职业道德高尚的汕尾工匠。

三、全力稳定有效投资，加快蓄积发展动能

——加大项目投资。以大投资促进大发展，聚焦关键领域和薄弱环节，谋划一批优质重大项目列入国家和省规划盘子，建立项目建设和财政投资相匹配的接续有序、滚动实施机制，争取三年内实施571个政府投资项目、完成投资超过2400亿元。持续优化投资结构，促进工业投资回升，稳定基础设施投资，保持房地产投资合理增长，引导更多资金投向强基础、增后劲、惠民生领域。健全重点项目建设推进机制，今年安排实施市重点项目189个，年度计划投资358亿元。实施工业投资三年行动计划，完成工业投资253亿元，推动27个产业项目开工建设，其中亿元以上项目24个。加快陆丰核电、海上风电、海工装备基地、比亚迪红草基地、比亚迪试车场、信利二期、威翔航空、海王医药、路华电子、国信通等一批产业项目建设，力争投产项目达21个以上。

——推动产业园区发展壮大。投入开发建设资金11亿元，推动园区征地5800亩，加快路水电气讯、标准厂房等基础设施建设，完善生活配套服务设施。对标深圳湾科技生态园，升级园区管理运营理念，与深投控合资共建“红海智谷”项目，打造园区运营新平台。集中优势兵力抓招商引资，采取以商招商、产业链招商等方式，落实“一把手”带队、点对点敲门式精准招商。围绕新落地投产的比亚迪、信利、香雪制药等龙头企业，吸引电子信息、新能源汽车、新材料、生物医药等产业链上下游企业集聚，力争引进投资5000万元以上项目50个。打好乡贤牌，办好第八届粤东侨博会，发挥珠三角、港澳台及海外200多万乡贤、3万多家乡贤企业的资源优势，吸引乡贤回乡投资兴业，利用乡贤人脉资源营销推介汕尾。

——推进现代化基础设施建设。投入37.8亿元，加快铁公机港交通基础设施建设，构建现代立体综合交通体系。推动广汕高铁、汕汕高铁加快建设，提升厦深铁路城际功能，加快深汕高铁、龙汕铁路前期工作。加快兴汕高速建设，确保年底前海丰至红海湾段一期基本具备通车条件，适时启动二期建设。推动深汕西高速公路改扩建，加快广东滨海公路汕尾段前期工作。动工建设珠东快速路，加强市区与陆丰、海丰的快速连接。新改建国道228线碣石至乌坎、324线海丰县城至梅陇、235线陆河县城至河口等一批国省道，力争新改建普通公路80公里。启动汕尾新港区、陆丰港区湖东作业区前期工作，恢复汕尾至香港海上客运航线，开展汕尾机场前期工作。启动公平水库至市区输水工程、市区备用水源、龙潭灌区节水改造、红草园区排洪治涝二期工程，推进赤沙水厂、水闸除险加固工程建设。推进新一轮信息基础设施建设三年行动计划，加快光纤宽带扩容提速，完善4G网络，推动5G网络试点建设，打造精品移动物联网。加快变电站、汽车充电桩、天然气管网建设。

——全力保障投资。落实好海陆丰革命老区振兴发展规划，用足用活财政、投资、产业、金融、土地、人才等六个方面59项扶持政策，加快推动列入规划、总投资达2700亿的30个项目开工建设。积极争取更多政府债券额度，做好政府债券项目实施准备，加快债券资金支出进度，确保形成实物工作量。加大投融资改革，组建市投控公司，探索投融资平台市场化转型。拓宽融资渠道，利用PPP等模式，鼓励引导民间资本通过市场化运作方式参与投资。加强土地收储，为产业发展和城市建设留足空间。加大对批而未供和闲置土地处置力度，力争全年消化10500亩。

四、大力实施创新驱动发展战略，增强经济质量优势

——推进科技创新载体建设。推进汕尾高新区申报国家级高新区，推动陆河新河工业园、海丰生态科技城申报省级高新区。积极推进惠州现代物理与清洁能源省实验室汕尾分中心建设。加快市产品质量检测公共技术平台建设。推进科技企业加速器、孵化器、众创空间、新型研发机构、企业研发中心等载体建设，积极对接大湾区和国内外高校、科研机构资源，共建产学研技术联盟、产业联盟、标准联盟等平台，力争新建省级工程技术研究中心8家，各类新型研发机构、研发中心、实验室等25家。

——提升自主创新能力。大力培育高新技术企业，新增国家高新技术企业15家以上。鼓励企业加大研发投入，力争R＆D经费占GDP比例达1.1%，新增专利申请量4800件、授权量3000件，PCT专利申请15件以上。鼓励企业以“机器换人”和“上云上平台”为重点实施技术改造，力争实施重点技改项目100个，完成技改投资130亿元以上，新增机器人应用180台（套）以上。

——强化创新要素支撑。坚持以企业为主体实施省、市科技重大专项，引导各类创新要素向企业集聚。推广“信利创新”经验，扶持企业加快科技创新和成果转化。树立人才是第一资源意识，推进红海扬帆人才计划，鼓励企业引进培育高层次领军型创新人才，落实子女就学、住房补贴等人才政策。加大战略性新兴产业重点领域人才培养力度，培养一批本土高技能人才。落实普惠性财税政策，发挥科技创新基金作用，加大创新型企业扶持力度。建设科技型企业信用数据库，完善科技金融服务体系。

五、全面深化改革开放，持续释放发展活力

——统筹推进各领域改革。按照中央、省统一部署，按时高质量完成市县机构改革，市县机构要利用改革调整契机，科学制定机构“三定”方案，优化内部职能和流程。落实市委关于加快推进新时代全面深化改革的若干意见。深化供给侧结构性改革，按照分类处置方式加快特困国有企业重组。深化财税体制改革，探索建立市级政策性担保体系。深化教育领域综合改革，完善城乡义务教育一体化优质均衡发展机制。发挥改革试点示范带动作用，抓好农村综合性改革、农村集体产权制度改革等试点工作。

——优化提升营商环境。深化“放管服”改革，发挥商事制度改革效应，全面实行市场准入负面清单制度，开展“减证便民”行动，推进“马上办、网上办、就近办、一次办”审批服务改革。加快推进“数字政府”建设，上线“粤省事”汕尾版，实现一站式“指尖办理”。出重拳解决各类“吃拿卡要拖”“潜规则”等“中梗阻”“下梗阻”问题，畅通行政审批、政府服务“最后一百米”，打造便捷高效的政务环境。推进工程建设项目审批制度改革，将审批时限压减至100个工作日以内。加强政府项目招投标、投资评审、政府采购、土地出让管理，加大对肉菜海鲜、煤气和建筑材料市场监管力度，建立工程建设企业短名单、黑名单制度，打造公平有序的市场环境。构建跨部门、跨领域的守信激励和失信惩戒机制，在政府采购、工程招投标等重点领域强化信用信息应用，打造诚实守信的信用环境。

——全面提高对外开放合作水平。落实省、市“外资新十条”，加大对外资企业市场准入、财政奖励、用地保障、金融服务等支持力度。落实深莞惠+汕河“3+2”经济圈联席会议重点合作事项。培育壮大民营出口企业，大力发展一般贸易，促进加工贸易转型升级。推进汕尾港口岸海丰小漠港区、陆丰甲湖港区对外开放，加快埔边快捷通关平台建设，提升“一网通办”通关服务，推进大通关一体化。

六、强化城市规划建设管理，系统提升城市品质

——拓展城市发展空间。对标广州、深圳等城市，高水平编制面向2035年的“多规合一”城市空间规划。统筹推进中心市区“东拓、西延、北扩、中优”，进一步拉开城市框架，重点加快中央商务区建设，推动市区与海城、红海湾同城化一体化发展。红海湾要重点开发田寮湖片区、白沙湖盐田片区、田墘新区，加快推进国家级海洋公园、滨海运动小镇建设，打造国际旅游休闲度假基地。海丰要推动海城、附城、城东组团式发展，加快与市区同城化，推进红城文旅小镇建设，打造“东方红城”。陆丰要加快市区扩容提质，向螺河两岸拓展，完善中等城市基本框架。陆河要推进陆河大道片区、“一河两岸”规划建设，建设客家新山城。华侨要围绕“一城两园”，打造岭南精品水果之乡。

——优化提升城市品质。从提升城市的质量品位着手，以规划为先导，统筹推进市政基础设施建设。畅通市区对外交通网络，高标准升级改造汕尾大道中（西闸至埔边）。推动工业大道西开工建设，打通盘活市区西北片区。以环品清湖至金町湾片区为核心区，完成环湖路景观提升，高品质建设海滨大道西及沿线区域。加快文明路、兴华路、翠园街等综合升级改造，打通金鹏路西段等10条断头路，推进青山仔、金台山等城市公园建设。完成奎山湖、奎山河整治，分批加快老城区雨污分流改造，优化提升自来水管网、天然气管道、防涝设施等城市配套功能。新投放300辆新能源出租车，推进华师附中北侧等4个公共停车场建设，全面整治市区乱停乱放，切实缓解中心城区“出行难、停车难”问题。高标准完成品清湖环湖灯光夜景工程。推进保利金町湾旅游休闲度假区、星河湾、恒大、雅居乐、华润万象天地、碧桂园商业中心、万达广场、万升广场、农商行大厦、信利中央广场、雍悦城和海丰华耀城、世纪广场、陆河华侨城新时代广场、岁宝广场等项目建设。推动和顺上村等一批“三旧”改造。开展城市艺术规划，打造彰显汕尾城市风貌和人文精神的雕塑公园。推进市民服务广场、“三馆一中心”（文化馆、博物馆、图书馆、演艺中心）、档案馆、少年宫、工人文化宫等公共设施建设。

——加强城市精细化管理。将“精细化”贯穿城市规划建设管理全过程，管好管优中心城区，向东涌、马宫等镇村延伸拓展。统筹考虑天际线、道路桥梁、灯光照明等城市风貌，提高市政设施、园林绿化的建设管养水平，提升主干道和重点区域整体景观效果。严格建筑立面管理，禁用马赛克、限用瓷砖、优选涂料。实施垃圾分类管理。保持打击违建高压态势，加大老城区、城乡结合部等重点区域违法建设整治力度，坚决遏制新增违法建设。加强城市数字化、网格化、常态化管理，推动城管执法重心下移。

——凝心聚力打好“创文”“创国卫”攻坚战。把巩固提升“创文”工作作为打造沿海经济带靓丽明珠的重要内容，紧紧围绕文明城市测评体系标准要求，狠抓工作落实，保持“创文”工作的常态化长效化。巩固提升省卫生城市成果，实施“创国卫”八大攻坚行动，推进示范区、示范街、示范小区等建设，确保年底前基本达标，力争2020年成功创建国家卫生城市。

七、扎实推进乡村振兴战略，加快农业农村现代化

——全域建设生态宜居美丽乡村。制定出台市乡村振兴战略规划，实现村庄建设规划全覆盖。加快推进全域人居环境整治，确保年底前所有自然村全面完成“三清理、三拆除、三整治”任务，60%以上自然村达到干净整洁村标准。打造美丽宜居乡村示范点38个，以点带面、串珠成链推动连片美丽乡村建设。推动垃圾、污水、供水、电网和网络等农村基础设施提档升级，加快166公里“四好农村路”建设。按照分步规划、分类施策，推进农村“厕所革命”，加快乡村旅游区等公共场所A级以上厕所、自然村标准化公厕建设。加强农村宅基地和农房建设管理，执行“一户一宅”政策和宅基地用地标准，引导农民集中建房，促进节约集约用地。

——大力发展富民兴村产业。依托现代农业产业园，引导优质特色农产品向生产优势产区集中，发展“一村一品、一镇一业”，做优传统产业、做大新兴产业、做强特色产业。争创一批国家级、省级农业标准示范区，推动一批农业优势产品创建国家地理标志保护产品。推进一二三产业融合发展，合理布局种养、加工、营销、休闲观光等农业产业，建设一批集农资储存配送、农产品检测冷链销售、农技推广、农民技术培训于一体的区域性“三农”综合服务平台。推进主要农作物生产全程机械化。推动撂荒土地复耕，耕地撂荒率控制在4%以下。开展红色展陈提升行动，完善景区配套设施建设，策划打造一批红色旅游精品景区景点，推动与生态旅游、滨海旅游、民俗文化旅游等融合发展。

——全力打好精准脱贫攻坚战。坚持精准扶贫、精准脱贫基本方略，主动对接深圳精准结对帮扶，确保1.7万相对贫困人口如期高质量脱贫。推广陆河螺洞“世外梅园”、海丰新山等经验，利用生态优势和资源禀赋发展乡村旅游，打造脱贫攻坚新亮点。推广“保底收益+按股分红”帮扶模式，鼓励引导贫困户入股收益项目，增加财产性收入。推广“公司+基地+贫困户”帮扶模式，引导有劳动能力的贫困户参与产业化经营，带动贫困户脱贫致富。全面落实就业扶贫、教育补助、医疗救助、危房改造、兜底保障、生态补偿等政策，巩固脱贫成果，防止返贫。

八、坚持绿色发展，全力保护生态环境

——加强生态环境保护。坚决抓好中央、省环保督察整改，严防反弹回潮。加强海河湖（库）岸线管理，实行海岸建筑退缩线制度，重点整治非法高位养殖、非法采砂、乱排污等破坏岸线问题。推动近岸海岛群保护开发利用，加快推进龟龄岛、遮浪岩等项目建设。强化生态空间管控，引导城镇建设、资源开发、产业发展合理布局。实行更加严格的土地管理制度。探索新建商品房绿化配套主体责任制。制定三年、五年、十年山体绿化行动计划，每年绿化一片，久久为功，十年功成，重点打造海汕公路、环品清湖、汕尾火车站及红海湾大道周边山体绿化景观。加快创建国家园林城市。严厉打击破坏山体行为，造林43万亩、森林抚育64万亩，创建乡村绿化美化示范点64个。

——打赢污染防治三大战役。实施水污染防治行动计划，开展全市45条、903公里中小河流专项整治，持续改善黄江河水质。强化集中式饮用水源区保护，保障群众饮水安全。推进蓝色海湾整治，加快品清湖海湾综合治理和生态岛礁建设。加强空气质量监测，全面治理“散乱污”工业企业等污染源，管控城市扬尘，确保空气质量继续位居全省前列。实施土壤环境动态监控，加强农业面源污染防治，推广测土配方施肥技术。实施受污染土地环境风险评估和修复。

——补齐环保基础设施短板。推进市生活垃圾无害化处理中心二期建设，完成30个镇级简易垃圾填埋场整改，实现生活垃圾处理“全焚烧”。打好生活污水处理设施和管网建设大会战，加快市东区水质净化厂建设，提标改造西区污水处理厂，实现建制镇污水处理设施全覆盖，新建污水管网100公里。加快星都循环经济产业园、工业固体废物处置中心建设，建成市医疗废物无害化集中处理中心。加强危险废物监管。

九、坚持以人民为中心，着力保障和改善民生

——优先发展教育事业。加大教育投入，全力推进教育“创现”，确保年底前实现教育现代化先进县（市、区）全覆盖。新改扩建幼儿园15所，公办幼儿园和普惠性民办幼儿园占比提高到80%以上。消除义务教育“大班额”现象，新改扩建义务教育阶段学校21所。扎实推进普通高中提质升级，加快彭湃中学、龙山中学、华美学校等学校建设，推进市林伟华中学创建国家级示范性高中。探索基础教育集团化办学，增加优质学位供给。大力发展职业技术教育，中等职业教育在校生占高中阶段教育在校生比例力争提高到35%，组建职校联盟，加强校企、校际交流合作，为企业源源不断输送高素质人才用工。加快市高级技工学校、汕尾职业技术学院新校区建设，办好开放大学，积极引进本科院校，提升特殊教育水平，促进民办教育规范健康发展。深化教师“县管校聘”、校长职级制等改革，落实中小学教师退出机制，加大校长选聘力度，加强师德师风建设，健全教育、宣传、考核、监督、奖惩相结合的师德师风建设长效机制。

——加快发展医疗卫生事业。强化“三医联动”，深化公立医院改革。提升基层医疗服务能力，大力推进紧密型医联体建设，加快建立分级诊疗制度，做实家庭医生签约服务。高质量建设中山大学深汕中心医院，打造辐射周边区域的高水平现代化三甲医院。加快市第三人民医院、市妇幼保健院及9家县级公立医院建设，推动3家中心卫生院升级为县级医院，完成第二批399家村卫生站规范化建设。加强医疗人才队伍建设，引进重点学科带头人等高层次人才，抓好医德医风建设。支持民营医院发展，规范引导社会办医。完善妇幼保健体系，提升疾病防控能力。

——提升就业和社会保障水平。坚持就业优先战略和积极就业政策，力争实现城镇新增就业5万人、转移劳动力就业2.8万人。深入实施“粤菜师傅”工程，举办市首届“粤菜师傅”技能大赛，培训技能人才500名以上。加快市人力资源服务产业园、中心镇人力资源市场建设。实施全民参保计划，抓好企业职工基本养老保险扩面征缴，推进机关事业单位养老保险制度改革，完善大病保险制度。完善社会救助体系和养老服务体系，推进老龄事业发展。发展住房租赁市场，新建一批限价房和租赁房。加快市福利中心、镇级公益性公墓山（骨灰楼）建设。

——繁荣发展文化体育事业。做好市城区省级新时代文明实践中心试点工作。实施基层文化馆（站）达标升级工程，实现行政村（社区）综合性文化服务中心全覆盖，启动沙坑文化博物馆规划建设，做好坎下城保护修缮。擦亮“中国民间文化艺术之乡”品牌，加强“四戏一歌”等非遗保护传承，培育一批特色文化骨干企业。办好第三届广东（汕尾）马思聪杯小提琴邀请赛。加快红色资源整体保护利用总体规划编制，抓好海陆丰革命根据地历史展览馆陈列布展、海陆丰革命烈士暨革命斗争史纪念馆展览升级、周恩来渡海处修缮改造等9个红色革命遗址保护利用项目的建设。启动建设海陆丰干部学院，精心打造红色教育基地。加快市体育运动学校和业余体校新校区建设，推进省海上运动中心升级改造，建设一批公共体育设施和社区体育公园。广泛开展全民健身运动，办好2019年汕尾马拉松赛事和市职工运动会。

集中力量办好省、市民生实事。

十、加快构建共建共治共享社会治理格局，营造安全稳定发展环境

——营造平安和谐稳定大局。全力维护国家政治安全和社会大局稳定，持续开展以打击涉盗抢、制假贩假、走私贩私、电信诈骗、地下钱庄、“套路贷”等违法犯罪为重点的专项行动。纵深推进扫黑除恶专项斗争，集中攻坚，深挖彻查，铲除黑恶势力滋生土壤，破除“关系网”、打掉“保护伞”、斩断“利益链”，做到不留死角、全面起底、依法打击。继续保持对毒品问题严打高压态势，巩固“摘帽”成果，坚决防止死灰复燃，创建全国禁毒工作示范市，加快戒毒所、戒毒医院建设。创建全国基层反走私综合治理示范市。推进雪亮工程、智慧新警务建设。完善“中心+网格化+信息化”综治工作体系，实现市县镇村视联网全覆盖。加强和改进信访维稳工作，深化风险隐患排查化解，解决重点领域、重点利益诉求群体突出矛盾。打好防范化解重大风险攻坚战，加快推进三家农信社改制组建农商行，严厉打击恶意逃废债行为，进一步压降农合机构不良贷款，做实捐赠资产。持续开展非法集资、互联网金融风险专项整治。促进房地产市场平稳健康发展，避免大起大落。

——推进公共安全体系建设。落实安全生产部门监管责任和企业主体责任，全面提高安全生产和应急管理能力，构建统一领导、权责一致、权威高效的应急救援体系，抓好城市风险点、危险源排查整治和重点行业领域隐患排查治理，坚决遏制重特大事故发生。加强工业产品、特种设备安全监管。实行最严格的食品药品安全监管制度，抓好校园、农村、食用农产品检测等专项整治。加强非洲猪瘟防控。加强地质、气象灾害监测预警，推进基层“三防”标准化建设，全面提升防灾减灾救灾能力。

——加强基层社会治理创新。实施基层基础保障工程，加强基层党组织建设。推进创新城乡社区治理专项改革试点。厘清基层权责边界，建立以协商议事制度为重点的多元治理机制，支持和鼓励工青妇等群团组织发挥桥梁纽带作用，推动各类社会组织参与社会治理。加强社工人才队伍和志愿者队伍建设，创新网格化管理和服务，完善社区服务设施，加强社区文化建设，推动“互联网+政务服务”体系延伸到城乡社区，提高社区政务服务和公共管理水平。

同时，继续做好军民融合发展、国防动员、人民防空、双拥共建、优抚安置等工作。做好民族、宗教、侨务、地震、档案、方志等各项工作。

做好今年工作，必须坚持和加强党的全面领导，坚决落实市委各项决策部署，全面加强政府自身建设，以实际成效开创政府工作新局面。一是加强法治政府建设。严格遵守宪法和法律。自觉接受人大监督，依法向市人大及其常委会报告工作。支持市政协履行政治协商、民主监督、参政议政职能。加强与各民主党派、工商联、无党派人士和各人民团体的沟通协调。认真办好人大代表建议和政协提案。严格落实重大行政决策和规范性文件合法性审查制度，强化重大决策终身责任追究及责任倒查。推进政务公开和依法行政，加强政府立法。二是加强廉政建设。认真落实《中国共产党纪律处分条例》，严格落实党风廉政建设各项规定，自觉接受巡视监督和监察委员会的监督，切实把权力关进制度的笼子。巩固发展反腐败斗争压倒性态势，深化标本兼治，加快形成不敢腐、不能腐、不想腐的体制机制。强化司法、审计监督，规范权力运行。三是全面加强作风建设。落实中央八项规定及实施细则精神，坚决整治各种隐形变异的“四风”问题和庸政懒政怠政。树立过“紧日子”思想，该减的支出一定要减下来，严控“三公”经费预算和一般性支出。建立健全激励机制和容错纠错机制，突出主责主业，教育广大干部学习新知识、掌握新本领，保持苦干实干、能干善干的实践品格，提高抓大事谋大事的能力水平。

雄关漫道真如铁，而今迈步从头越!各位代表，让我们更加紧密团结在以习近平同志为核心的党中央周围，坚持以习近平新时代中国特色社会主义思想为指导，在省委、省政府和市委坚强领导下，发扬海陆丰革命老区敢为人先的革命精神，闻鸡起舞、日夜兼程、风雨无阻，鼓足勇气迎难而上，提振精神开拓进取，奋力把汕尾建设成为沿海经济带的靓丽明珠，为广东实现“四个走在全国前列”、当好“两个重要窗口”贡献汕尾力量，以优异成绩庆祝中华人民共和国成立70周年!
